# Disrupting GluA2-GAPDH Interaction Affects Axon and Dendrite Development

**DOI:** 10.1038/srep30458

**Published:** 2016-07-27

**Authors:** Frankie Hang Fung Lee, Ping Su, Yu-Feng Xie, Kyle Ethan Wang, Qi Wan, Fang Liu

**Affiliations:** 1 Campbell Family Mental Health Research Institute, Centre for Addiction and Mental Health, Toronto, Ontario, M5T 1R8 Canada; 2 Department of Physiology, School of Medicine, Wuhan University, Wuhan 430071, China; 3 Department of Psychiatry, University of Toronto, Toronto, Ontario, M5T 1R8 Canada

## Abstract

GluA2-containing AMPA receptors (AMPARs) play a critical role in various aspects of neurodevelopment. However, the molecular mechanisms underlying these processes are largely unknown. We report here that the interaction between GluA2 and glyceraldehyde 3-phosphate dehydrogenase (GAPDH) is necessary for neuron and cortical development. Using an interfering peptide (GluA2-G-Gpep) that specifically disrupts this interaction, we found that primary neuron cultures with peptide treatment displayed growth cone development deficits, impairment of axon formation, less dendritic arborization and lower spine protrusion density. Consistently, *in vivo* data with mouse brains from pregnant dams injected with GluA2-G-Gpep daily during embryonic day 8 to 19 revealed a reduction of cortical tract axon integrity and neuronal density in post-natal day 1 offspring. Disruption of GluA2-GAPDH interaction also impairs the GluA2-Plexin A4 interaction and reduces p53 acetylation in mice, both of which are possible mechanisms leading to the observed neurodevelopmental abnormalities. Furthermore, electrophysiological experiments indicate altered long-term potentiation (LTP) in hippocampal slices of offspring mice. Our results provide novel evidence that AMPARs, specifically the GluA2 subunit via its interaction with GAPDH, play a critical role in cortical neurodevelopment.

AMPA-type receptors (AMPARs) are the primary mediators of fast excitatory synaptic transmission in the mammalian central nervous system, and are crucial in regulating higher brain functions such as learning and memory^1^,^2^. They exist as heteromeric combinations of four subunits, namely GluA1–4^3^. All subunits can have significant impact on AMPAR properties or functions. But in particular, GluA2 subunits are the most crucial determinants in controlling the biophysical properties of calcium permeability, receptor kinetics and channel conductance^1^. During early development, GluA2 expression is strictly regulated by RNA editing and alternative splicing, which determine receptor properties as well^4^,^5^,^6^. Moreover, phosphorylation of AMPARs, mainly mediated by protein kinase A, C, Ca^2+^/calmodulin-dependent protein kinase II and tyrosine kinases^7^, and specific GluA2-interacting partners provide distinct control of receptor functions^1^,^2^. For example, the C-terminal domain of GluA2 has been exclusively studied for its association with glutamate receptor-interacting protein (GRIP)^8^ and protein interacting with C-kinase 1 (PICK1)^9^ in regulating AMPAR trafficking and endocytosis. Transmembrane AMPAR regulatory proteins (TARPs) such as stargazin^10^, CKAMP proteins (Shisa9)^11^,^12^,^13^ and a family of cornichon proteins^14^ have all been identified acting as auxiliary subunits and interact with GluA2 to modulate AMPAR functions. Neuronal activity-regulated pentraxin (Narp) has been shown to interact with the N-terminal domain (NTD) of GluA2, playing a role in AMPAR clustering which may link to development and plasticity of excitatory synapses^15^. Moreover, recent research demonstrated that this domain of GluA2 can also interact with N-cadherin, a cell adhesion protein, and Plexin A receptors, responsible for semaphorin-mediated neuron guidance signaling, where they play important roles in spine formation and dendritic development, respectively^16^,^17^.

In our previous studies, we have identified a novel interaction between GluA2 and glyceraldehyde 3-phosphate dehydrogenase (GAPDH), and that an enhanced complex formation is associated with neuronal cell death^18^,^19^,^20^. GAPDH was shown to interact with p53 which subsequently promoted this cell death pathway via p53 phosphorylation^21^. This provided new insights about the cell signaling properties of the GluA2-GAPDH interaction. Interestingly, recent reports have demonstrated that p53 acetylation of lysine residues regulates neurodevelopmental processes^22^,^23^. As GluA2 subunits are also essential in various aspects of neurodevelopment and we observed the existence of GluA2-GAPDH interaction in cells/neurons without glutamate stimulation and post-mortem spinal cord tissues of healthy human subjects, it is likely that this interaction is crucial under physiological conditions in regulating proper development^19^. Therefore, we investigated the functional consequences of disrupting the GluA2-GAPDH interaction using an interfering peptide on neuronal growth and cortical development.

Based on *in vitro* and *in vivo* data, we demonstrated that GluA2-GAPDH disruption results in axon integrity defects, less dendritic branching, reduced spine protrusion density and fewer total neuron numbers. These histological deficits may at least be partly mediated via a reduction in GluA2-Plexin A4 interaction and p53 acetylation. Furthermore, we observed altered long-term potentiation (LTP) in hippocampal slices of peptide-treated offspring mice. Our data provides novel information on the role of GluA2-GAPDH interaction in neurodevelopment.

## Results

### Disrupting GluA2-GAPDH interaction leads to deficits in growth cone and axon development in primary neuron cultures

**Figure 1:**
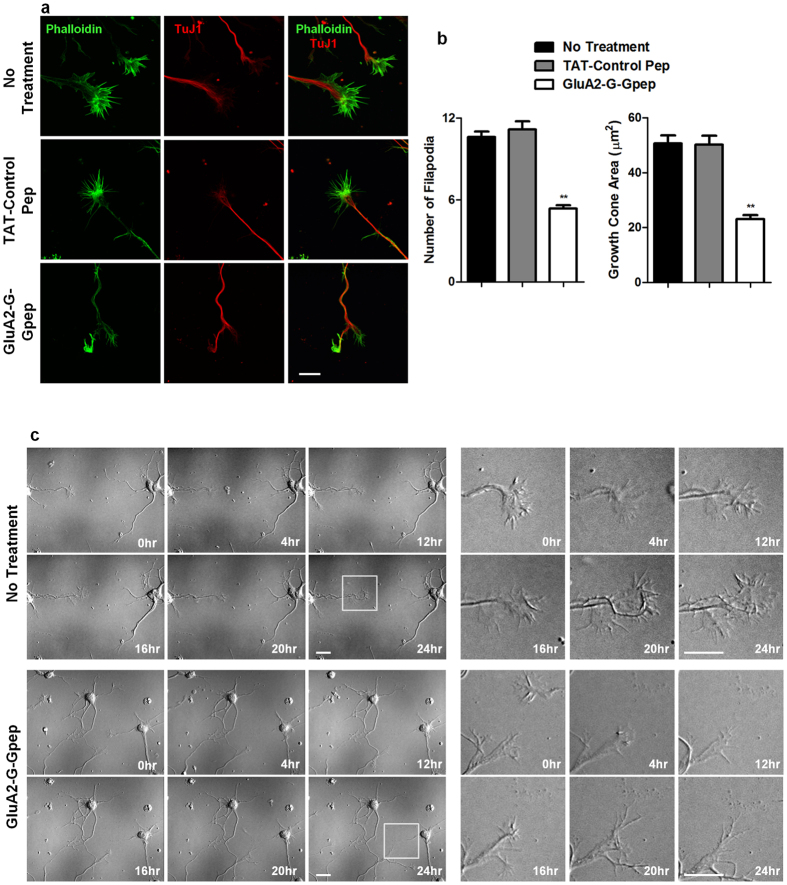
GluA2-G-Gpep treatment leads to growth cone collapse in primary cultured neurons. (**a**) Representative immunofluorescent images showing axonal and dendritic growth cones in non-treated, TAT-control pep- and GluA2-G-Gpep-treated (10 μM) primary neurons at 3 days *in vitro* (DIV). Scale Bar: 10 μm. (**b**) The number of filapodia and growth cone area of GluA2-G-Gpep-treated neurons (n = 93 neurons from 4 cultures) was significantly less than those observed in control neurons (No treatment: n = 105 neurons; TAT-Control pep: n = 86 neurons from 4 cultures; two-tailed *t*-test). (**c**) Time-lapse imaging of growth cone development showed that primary neurons at 3 DIV with GluA2-G-Gpep treatment had smaller growth cone areas and showed less filopodia dynamics when compared to control. Scale Bar: 10 μm. Data are presented as mean ± SEM. **p < 0.01.

**Figure 2:**
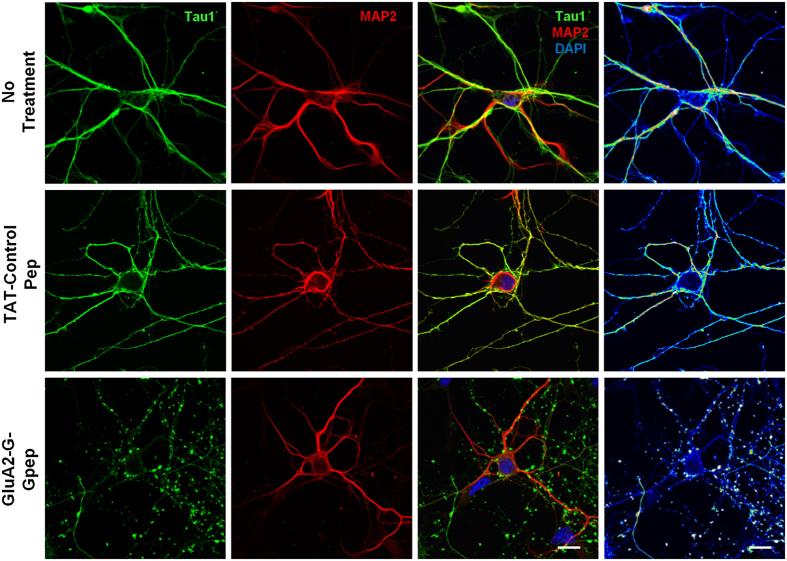
Axon development is impaired with GluA2-G-Gpep treatment in primary neurons. Immunostaining was performed with Tau1 and MAP2 antibodies in primary neurons at 8 DIV with no treatment, TAT-control peptide treatment and GluA2-G-Gpep treatment. Heat intensity map of Tau1 fluorescence showed disintegrated Tau1-labeled axons with peptide treatment (n = 24 neurons per group from 3 cultures), while control neurons had a more evenly distributed Tau1 staining pattern. Scale Bar: 10 μm. Data are presented as mean ± SEM. **p < 0.01.

There is evidence for the involvement of GluA2 in early axon development^24^,^25^. Thus, we initiated our study by examining the effects of GluA2-G-Gpep treatment on growth cone dynamics in primary neuron cultures. Immunocytochemistry showed that dendritic and axonal growth cones of peptide-treated neurons had significantly less fluorescent staining of phalloidin (Fig. 1a). The number of filopodia per growth cone (No Treatment: 10.62 ± 3.87, n = 93; GluA2-G-Gpep: 5.36 ± 2.6; n = 105, two-tailed *t*-test, *p* < 0.01) and average growth cone area were profoundly reduced in neurons with peptide treatment as well (Fig. 1b). A TAT-control peptide was used to examine any peptide effects, but no significant difference was observed when compared to non-treated neurons (Fig. 1a,b). Time-lapse imaging of growth cone development was further performed on primary neurons, and consistently, peptide administration into neurons resulted in less filopodia movements (Fig. 1c). Impaired growth cone dynamics and growth cone collapse can lead to abnormal axon development^26^. To further elucidate the role of GluA2-GAPDH interaction in axonal growth, primary neurons were immunostained with Tau-1. Interestingly, GluA2-G-Gpep-treated neurons displayed an irregular Tau-1 staining pattern, characterized by non-uniform Tau-1 fluorescence scattered along developing axons. On the contrary, Tau-1 proteins were more evenly distributed in non-treated and TAT-control peptide-treated neurons, as shown in fluorescent intensity heat maps of Tau-1 immunostaining (Fig. 2). These results indicate that disruption of GluA2-GAPDH interaction can impair growth cone development, which in turn produce deficits in axon integrity.

### Reduced dendritic arborization and spine protrusion density in primary neurons pretreated with GluA2-G-Gpep

**Figure 3:**
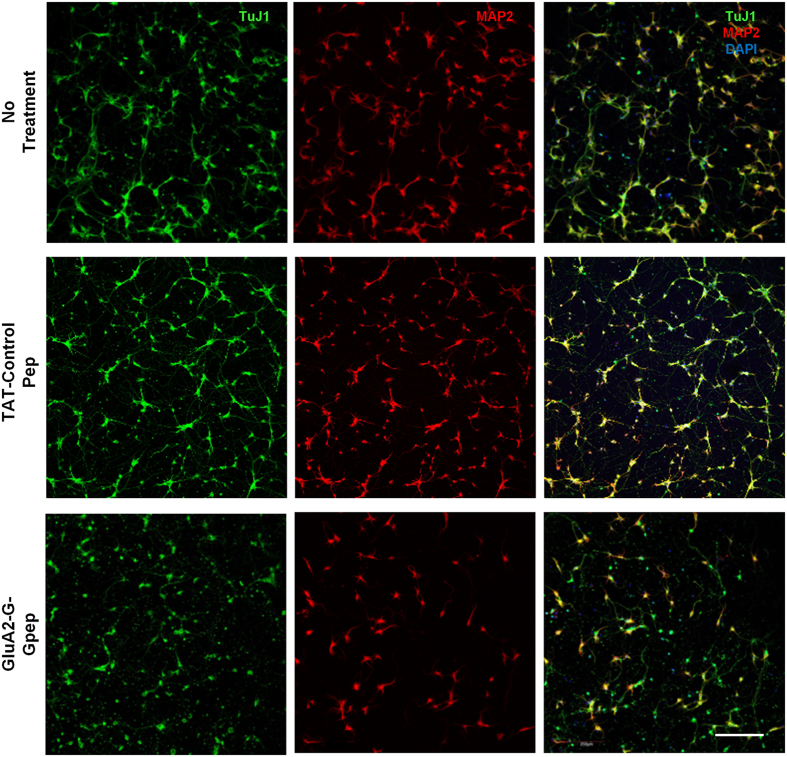
Disruption of GluA2-GAPDH interaction in primary cultured neurons results in fewer neurite branches and connections. Fluorescent images were captured at a low magnification of 10× to provide an overall view of neuronal cultures. Immunostaining of TuJ1 and MAP2 on 8 days *in vitro* (DIV) primary neurons revealed that GluA2-G-Gpep treatment (10 μM) had less connections within neurons compared to non-treated and TAT-control peptide-treated groups. Scale Bar: 200 μm.

**Figure 4:**
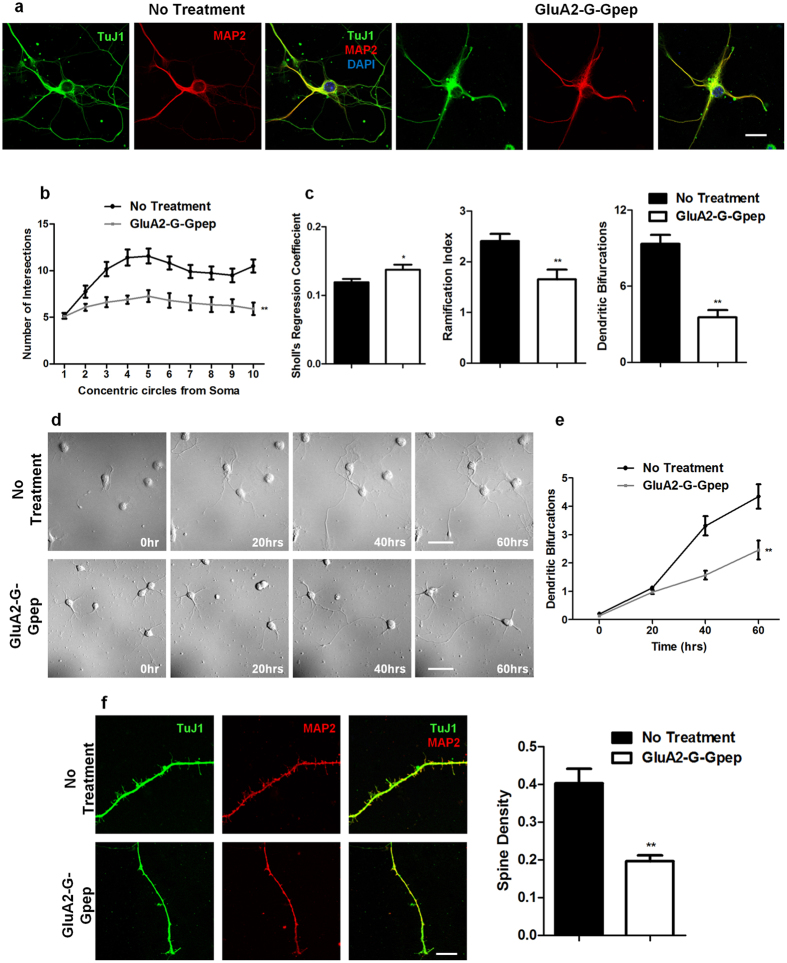
Reduced dendritic arbors and spine protrusion density in GluA2-G-Gpep-treated primary neurons. (**a**) Higher magnification images of individual neurons immunostained with TuJ1 and MAP2 were captured for dendritic branching analysis. Scale Bar: 20 μm. (**b**) There was a pronounced reduction of dendritic arbors in the GluA2-G-Gpep treatment group as measured by Sholl analysis (n = 30 neurons from 3 different cultures per group; one-way ANOVA, *post hoc* Bonferroni correction). (**c**) GluA2-G-Gpep-treated neurons also displayed a significantly higher Sholl regression coefficient, lower Ramification index and lower dendritic bifurcations (n = 30 neurons from 3 different cultures per group; two-tailed *t*-test). (**d**) Time-lapse imaging of primary neurons at 2 DIV revealed less complex neuronal branching patterns with GluA2-G-Gpep. Scale Bar: 50 μm. (**e**) Quantification of dendritic bifurcations in relation to growth time showed that neurons with peptide treatment developed significantly fewer dendritic branches. (n = 42 neurons from 3 different cultures per group; two-tailed *t*-test). (**f**) Spine protrusion density analysis on 12 DIV neurons showed significant reduction with GluA2-G-Gpep treatment when compared to controls (No Treatment: n = 31; GluA2-G-Gpep: n = 33 neurons from 3 different cultures; two-tailed *t*-test). Scale Bar: 5 μm. Data are presented as mean ± SEM. *p < 0.05; **p < 0.01.

Multiple studies have demonstrated that GluA2 is involved in regulating dendritic arbor formation^16^,^27^,^28^,^29^. We investigated whether disrupting normal GluA2-GAPDH interaction would alter dendritic complexity. An overall view of cultured neuron connections showed that peptide-treated neurons have substantially fewer connections than controls (Fig. 3). Next, we evaluated dendritic arborization at the individual neuronal level via Sholl analysis (Fig. 4a). Dendritic branching patterns were significantly less complex with increasing soma distance in GluA2-G-Gpep-treated neurons when compared to controls (Fig. 4b). In addition, peptide treatment resulted in a higher Sholl regression coefficient (No Treatment: 0.119 ± 0.018; GluA2-G-Gpep: 0.137 ± 0.024; n = 30, two-tailed *t*-test, *p* < 0.05), lower Ramification Index (No Treatment: 2.41 ± 0.5; GluA2-G-Gpep: 1.65 ± 0.64; n = 30, two-tailed *t*-test, *p* < 0.01) and reduced dendritic bifurcations (No Treatment: 9.33 ± 2.61; GluA2-G-Gpep: 3.55 ± 1.92; n = 30, two-tailed *t*-test, *p* < 0.01) (Fig. 4c), all of which indicated a lower dendritic complexity. To directly demonstrate that GluA2-GAPDH disruption can hinder neuritic branching, we used time-lapse imaging to capture the dendritic growth pattern of primary neurons at 2 days *in vitro* (DIV) (Fig. 4d). Neurons with peptide treatment had significantly fewer dendritic branches from the soma as measured by the dendritic bifurcations in relation to time (Fig. 4e).

The increase in dendritic spine density in hippocampal neurons with GluA2 overexpression reported by Passafaro’s group provided strong evidence for the association between GluA2 and spine development^17^,^30^. Hence, we investigated the role of GluA2-GAPDH complex on spinogenesis. Quantification of dendritic spine protrusion density revealed that peptide-treated neurons at 12 DIV had a marked 51% reduction versus control (No Treatment: 0.403 ± 0.176, n = 31; GluA2-G-Gpep: 0.196 ± 0.062, n = 33; two-tailed *t*-test, *p* < 0.01) (Fig. 4f). Together, these findings suggest that the interaction between GluA2 and GAPDH is essential for proper dendritic arbor formation and spine development.

### TAT-GluA2-G-Gpep peptide injection into pregnant mice results in cortical axon tract loss in the neonatal brain

Our next approach was to examine whether similar axon defects and other histological deficits would be observed when GluA2-GAPDH interaction is disrupted *in vivo*. To achieve this aim, we specifically injected pregnant mice with 5 nmol/g of GluA2-G-Gpep peptide daily from E8-E19, where neurodevelopment is most prominent. Our previous studies have extensively shown that this peptide significantly disrupts GluA2-GAPDH interaction and produces minimal peptide effect^18^,^19^,^20^. Recombinant proteins fused to TAT have been used extensively for efficient delivery of full-length functional proteins into animals *in vivo*, with great success in crossing the blood-brain barrier and placenta^31^,^32^. Here, we first confirmed that GluA2-G-Gpep applied under our treatment paradigm was capable of entering embryonic mouse brains. Immunohistochemistry against TAT showed more intense fluorescent signals in peptide-injected P1 brains than the saline group (Supplementary Fig. 1a), indicating a higher TAT-fusion protein concentration. Next, co-immunoprecipitation between GluA2 and GAPDH was performed with E16 brains to ensure the effectiveness of GluA2-G-Gpep in blocking this interaction. GluA2 antibody was able to co-immunoprecipitate with GAPDH in brain tissues, and this interaction was significantly reduced in peptide-treated brains as expected (Supplementary Fig. 1b). Total expression levels of GluA2 and GAPDH were similar between the two groups (Supplementary Fig. 1c). These results verified that GluA2-G-Gpep injection into pregnant mice can reach embryonic brains and disrupt the GluA2-GAPDH interaction.

**Figure 5:**
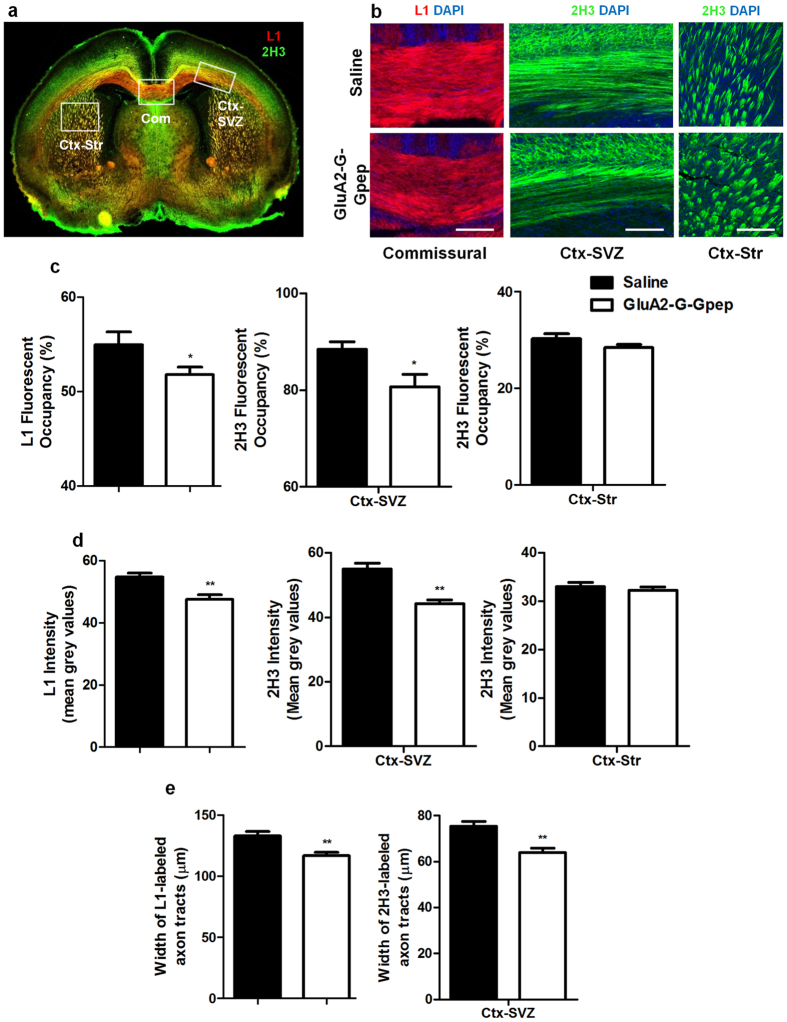
GluA2-G-Gpep administration disrupts axon integrity *in vivo*. (**a**) Immunohistochemistry of L1 and 2H3 was performed on P1 brain sections to detect and analyze cortical axonal tracts in three rectangular ROIs outlining the commissural tracts (Com), cortical tracts under subventricular zone (Ctx-SVZ) and cortico-striatal tracts (Ctx-Str). Scale Bar: 300 μm. (**b**) Higher magnification fluorescent images in the respective regions of interest (ROIs) are shown for both groups. Scale Bar: 50 μm. L1 and 2H3 staining was quantified using (**c**) a normalized thresholding scale and measured as percent area occupancy, and (**d**) mean grey values of fluorescent intensity with ImageJ. When compared to saline groups, GluA2-G-Gpep-treated mouse brains showed a significant decrease in L1-labeled commissural axon tracts, and 2H3-labeled cortical tracts beneath the subventricular zone. However, there were no significant changes in the cortico-striatal tracts between the two groups. (**e**) In addition, both the width of L1-commissural axon tracts and 2H3-cortical tracts in the SVZ were significantly smaller with peptide treatment (saline: n = 20; GluA2-G-Gpep: n = 24 brain sections from 4 different brains; two-tailed *t*-test). Data are presented as mean ± SEM. *p < 0.05, **p < 0.01.

We continued to investigate axonal tract development *in vivo* by immunostaining L1 and 2H3 on P1 neonatal brains. Axonal tract integrity was analyzed in three brain regions, including commissural axon tracts in corpus callosum (labeled by L1), cortico-cortical tracts beneath the subventricular zone (SVZ) of the cortex (Ctx-SVZ, labeled by 2H3), and cortico-striatal tracts connecting to the striatum (Ctx-Str, labeled by 2H3) (Fig. 5a). Representative fluorescent images of L1 and 2H3 in selected regions of both groups are shown in Fig. 5b. Peptide treatment during early development resulted in significantly less L1-labeled commissural axons (saline: 54.92 ± 6.18%, n = 20; GluA2-G-Gpep: 51.79 ± 3.88%, n = 24; two-tailed *t*-test, *p* < 0.05) and loss of cortico-cortical axon tracts in the SVZ (saline: 88.47 ± 6.94%, n = 20; GluA2-G-Gpep: 80.71 ± 12.39%, n = 24, two-tailed *t*-test, *p* < 0.05) when compared to controls (Fig. 5c). However, axon intensity within the cortico-striatal circuit was not different between the two groups (Fig. 5c). Consistently, mean grey values of fluorescence intensity showed similar changes in all three regions (Fig. 5d), and that the width of both L1-commissural and 2H3-cortical tracts in the SVZ were significantly narrower with peptide treatment (Fig. 5e). These data further support a functional role of GluA2-GAPDH interaction in normal axon development, specifically in cortico-cortical connections.

### Cortical neuron density and neuronal proliferation is reduced in P1 mouse brains with GluA2-G-Gpep treatment

**Figure 6:**
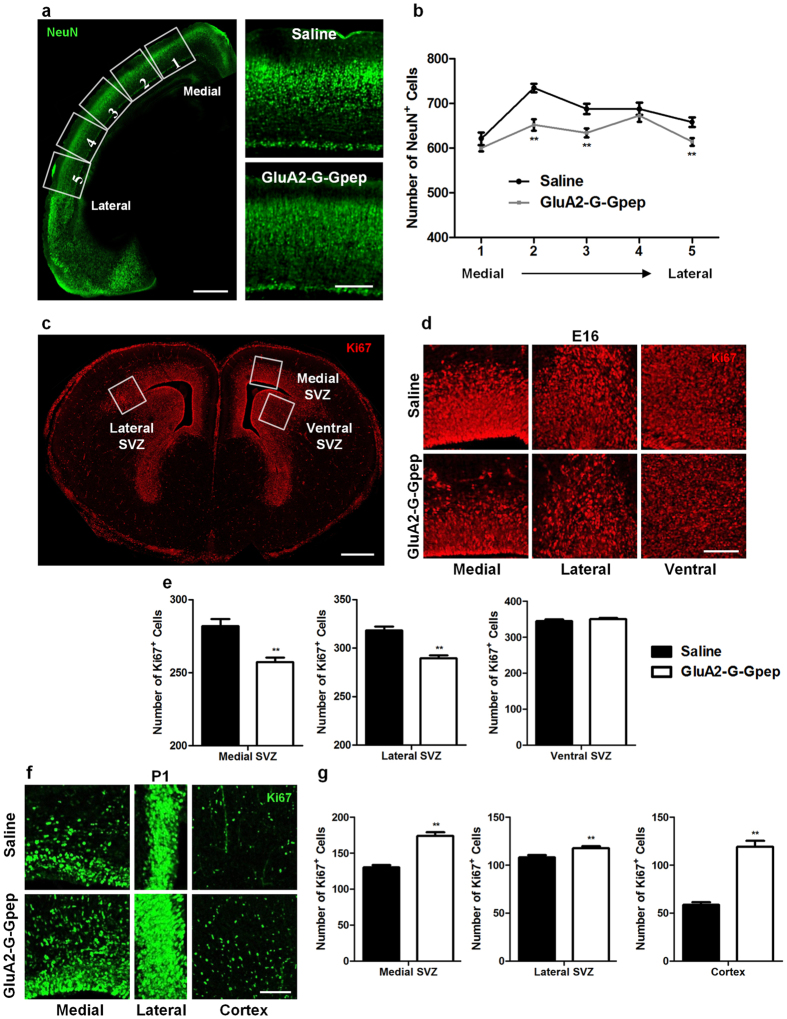
Reduced cortical neuron density with decreased neurogenesis in mice with GluA2-G-Gpep peptide treatment. (**a**) NeuN-immunostained images of the whole cortex were captured and delineated into five equal regions of interest (ROIs) along the medial-lateral axis. Scale Bar: 200 μm. Higher magnification images of NeuN-labeled cortex from saline and GluA2-G-Gpep groups are shown on the right. Scale Bar: 100 μm. (**b**) Quantification of NeuN^+^ cells showed that GluA2-G-Gpep treatment resulted in significantly fewer neurons across the cortex and lower total neuron number (saline: n = 21; GluA2-G-Gpep: n = 24 sections from 3 different brains; one-way ANOVA, *post hoc* Bonferroni correction; two-tailed *t*-test). (**c**) Neuronal proliferation was detected using immunohistochemistry against Ki67. Fixed regions positioned over the medial, lateral and ventral subventricular zone (SVZ) for analysis are outlined with E16 coronal brain sections. Scale Bar: 300 μm. Representative fluorescent images of Ki67-immunostained cells in (**d**) E16 and (**f**) P1 saline and GluA2-G-Gpep-treated mouse brains. Scale Bar: 50 μm. (**e**) For E16 brains, there were significantly fewer Ki67^+^ cells in the medial and lateral SVZ of GluA2-G-Gpep treatment brains when compared to controls, while no difference was observed in the ventral SVZ (saline: n = 30; GluA2-G-Gpep: n = 26–27 sections from 3 different brains; two-tailed *t*-test). (**g**) On the contrary, P1 neonatal brains treated with GluA2-G-Gpep showed a significant increase in Ki67^+^ cell numbers in all analyzed regions (medial SVZ, lateral SVZ and cortex) (saline: n = 28; GluA2-G-Gpep: n = 20 sections from 3 different brains; two-tailed *t*-test). Data are presented as mean ± SEM. **p < 0.01.

Axonal loss may associate with fewer neurons. Since we have given strong evidence for the role of GluA2-GAPDH interaction in neuronal cell death, we asked whether disrupting this interaction below physiological levels during neurodevelopment would affect neuronal density. The number of neurons throughout the neocortex along the medial-lateral axis of P1 brains was examined with NeuN, a neuronal marker (Fig. 6a). There were significantly fewer NeuN^+^ neurons across the neocortex in each region of interest (ROI), as well as total neuron numbers in GluA2-G-Gpep-treated mice compared to saline groups (Fig. 6b). To further elucidate whether this difference in neuron density could be related to altered neurogenesis, we assessed neuronal proliferation by immunolabeling a proliferation marker, Ki67. Three regions near the SVZ and cortex of E16 and P1 brain sections were chosen for analysis, where Ki67-positive progenitors are predominantly expressed (Fig. 6c,d). At E16, there were significantly fewer Ki67^+^ cells in the medial and lateral SVZ of GluA2-G-Gpep-treated brains when compared to saline (medial SVZ - saline: 282.03 ± 26.42, GluA2-G-Gpep: 257.23 ± 15.57; lateral SVZ - saline: 318.3 ± 21.6, GluA2-G-Gpep: 289.63 ± 15.69; n = 26–30, two-tailed *t*-test, *p* < 0.01), while no difference was observed in the ventral SVZ (Fig. 6e). In contrast, P1 neonatal brains with peptide treatment showed a marked increase in Ki67^+^ cell numbers in all analyzed regions (medial SVZ - saline: 130.36 ± 17.33, GluA2-G-Gpep: 174.1 ± 21.92; lateral SVZ - saline: 108.04 ± 12.7, GluA2-G-Gpep: 117.8 ± 8.19; ventral SVZ - saline: 58.62 ± 14, n = 28; GluA2-G-Gpep: 119 ± 28.32, n = 20, two-tailed *t*-test, *p* < 0.01) (Fig. 6f,g). These data suggest that blocking GluA2-GAPDH interaction below normal levels can also lead to reduced neuron numbers, possibly via affecting the neurogenesis process.

We also analyzed cortical neuron laminar distribution by using Cux1 and Ctip2 to label neurons specifically in cortical layers II/III and IV, respectively (Supplementary Fig. 2a,b). Interestingly, we observed a significantly higher proportion of Cux1^+^ cells positioned within superficial layers of octants 2 and 3 in GluA2-G-Gpep-treated mice, whereas the distribution in saline-treated mice was more widespread (Supplementary Fig. 2c). For Ctip2, GluA2-G-Gpep-treated mice displayed a shift towards deeper cortical layers with proportionally more cells in octants 6, 7 and 8, but less in octants 3 and 4 when compared to controls (Supplementary Fig. 2d). Our results indicate that the localization of cortical neurons was altered in mice with GluA2-G-Gpep treatment, suggesting additional roles of the GluA2-GAPDH interaction in cortical lamination. Further research is required to elucidate the underlying mechanisms in the relationship between the GluA2-GAPDH complex with neurogenesis and neuronal positioning.

### GluA2-GAPDH disruption reduces GluA2 interaction with Plexin A4 and expression levels of acetylated Lys320 of p53, but increases GluA2-stargazin complex formation in embryonic brains

**Figure 7:**
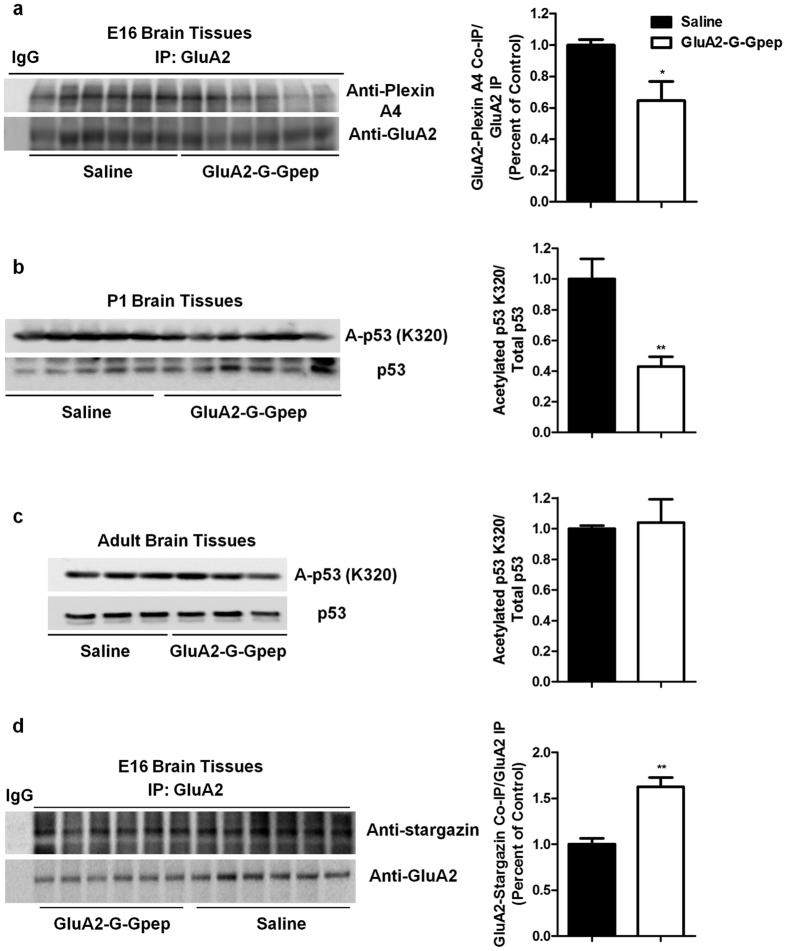
Disrupting GluA2-GAPDH interaction during early development leads to reduced GluA2-Plexin A4 interaction and expression levels of acetylated Lys320 (K320) of p53 but increased GluA2-stargazin complex levels in embryonic brains. (**a**) Co-immunoprecipitation of E16 brains showed a significant decrease in GluA2-Plexin A4 interaction with GluA2-G-Gpep treatment when compared to saline groups (n = 6 brains per group, two-tailed *t*-test). (**b**,**c**) Western blot analysis revealed a pronounced reduction of acetylated K320 levels in peptide-treated P1 brains when compared to controls (saline: n = 5; GluA2-G-Gpep: n = 6 brains, two-tailed *t*-test), but no significant difference in adult mice (n = 3 brains per group, two-tailed *t*-test). (**d**) In contrary, GluA2-stargazin complex levels were increased in peptide-treated mice as measured by co-immunoprecipitation. Quantification of GluA2 interaction with other proteins were normalized with GluA2 immunoprecipitation bands and expressed as a percentage of saline controls. Data are presented as mean ± SEM. *p < 0.05; **p < 0.01.

N-cadherin and Plexin A4 both interact with GluA2 at the NTD in regulating spine development and dendritic outgrowth^16^,^17^. It is possible that disrupting GluA2-GAPDH interaction would lead to alteration in its interaction with N-cadherin and Plexin A4, and subsequently resulting in neurohistological deficits. To explore this possibility, we quantified these interactions separately using co-immunoprecipitation on E16 brains with GluA2-G-Gpep treatment. Interestingly, peptide-treated groups displayed significantly less GluA2-Plexin A4 complex in E16 brains (Fig. 7a), but showed no difference in GluA2-N-cadherin interaction between the two groups (Supplementary Fig. 3a). Using a glutathione S-transferase (GST) pull-down assay, we verified that there was a direct interaction between GluA2-G-Gpep and GAPDH, but not with Plexin A4, thus eliminating a possible direct effect of our peptide on the GluA2-Plexin A4 interaction (Supplementary Fig. 3b). GST-FARP2 was used as a positive control for Plexin A4 interaction^33^.

Accumulating evidence suggests that post-translational modification of p53, specifically acetylation of lysine residues, can also modulate neurite outgrowth and axon regeneration^22^,^23^. Hence we examined expression levels of acetylated p53 K320 and K382 in GluA2-G-Gpep-treated P1 and adult mouse brains. As shown in Fig. 7b, there was a pronounced reduction of acetylated K320 levels per total p53 in peptide-treated P1 brains when compared to controls. Meanwhile, adult mice with peptide treatment displayed no change in acetylated K320 (Fig. 7c), and that acetylated K382 expression levels were comparable in all experimental groups, suggesting the specificity of this post-transcriptional modification of p53 (Supplementary Fig. 3c,d). In addition, we examined whether GluA2 interaction with auxiliary units, including stargazin and Narp, would be affected with GluA2-G-Gpep treatment. Surprisingly, we found a significant increase in GluA2-stargazin complex (Fig. 7d), but not with Narp (Supplementary Fig. 3e) when GluA2-GAPDH interaction was disrupted in early development. Finally, we performed immunohistochemistry on P1 brain sections labeling GluA2 with either Plexin A4 or acetylated p53, and found that all proteins are expressed and localized in the cortex (Supplementary Fig. 4). Altogether, these results indicate that GluA2-GAPDH disruption during development may specifically affect GluA2 interaction with Plexin A4 and stargazin, and the expression levels of post-translational modified p53. This may represent possible mechanisms in which how interfering the GluA2-GAPDH interaction can produce axon and dendritic defects.

### GluA2-G-Gpep-treated mice at 6 weeks old display lower spine density and altered synaptic activity

**Figure 8:**
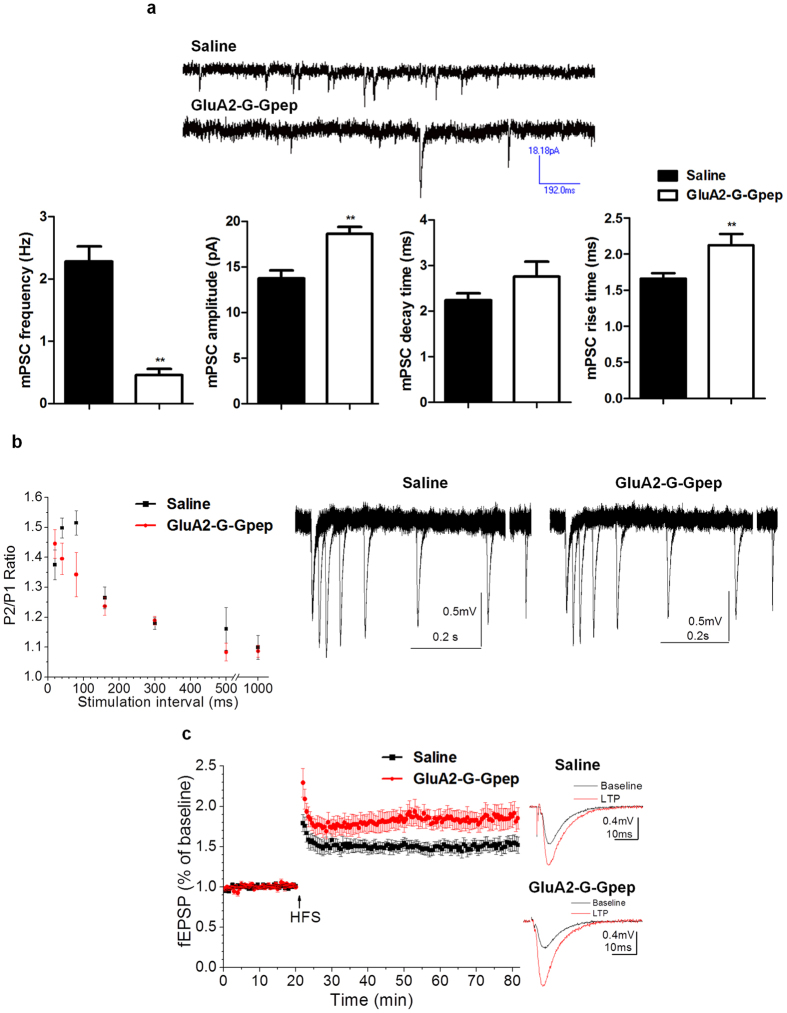
GluA2-G-Gpep treatment during early development produces alterations of synaptic activity in older offspring mice. (**a**) GluA2-G-Gpep treatment significantly decreased the frequency of mPSCs, increased mPSC amplitude and rise time, but had no effects on mPSC decay time when compared to controls (saline: n = 9; GluA2-G-Gpep: n = 14 brains; two-tailed *t*-test). (**b**) 6-week old mice with GluA2-G-Gpep treatment had no significant difference in paired-pulse ratio in field excitatory postsynaptic potential (fEPSP) recordings of Schaffer collateral-CA1 synapses. (**c**) Offspring mice with early developmental GluA2-G-Gpep treatment also displayed enhanced metaplasticity in Schaffer collateral-CA1 synapses. The left panel shows a plot of long-term potentiation (LTP) induced by high frequency stimulation (HFS, 100 pulses at 100 Hz in 1 s with repetition of 3 times at an interval of 30 s (saline: n = 6; GluA2-G-Gpep: n = 8 brains). Traces from saline and GluA2-G-Gpep treatment groups before (black, baseline) and after (red, LTP) HFS are shown in the right. Data are presented as mean ± SEM. **p < 0.01.

Since we have already shown that disrupting the GluA2-GAPDH interaction can alter an array of neurohistological features, we tested whether functional aspects of neuronal transmission would be impaired in 6-week old offspring mice with GluA2-G-Gpep treatment during developmental period. First, we performed a simple Golgi-Cox staining in these mice to measure dendritic spine density in cortical neurons (Supplementary Fig. 5a). GluA2-G-Gpep-treated mice had significantly lower spine density compared to saline groups (saline: 0.521 ± 0.106, n = 78; GluA2-G-Gpep: 0.426 ± 0.085; n = 88; two-tailed *t*-test; *p* < 0.01) (Supplementary Fig. 5b). Next, we measured neuronal functions based on different miniature postsynaptic currents (mPSCs) parameters in hippocampal slices (Supplementary Fig. 6). Consistent with the spine density changes induced by GluA2-G-Gpep, we found that the frequency of mPSCs in peptide-treated pyramidal neurons was significantly decreased when compared to saline (saline: 2.28 ± 0.245 Hz; GluA2-G-Gpep: 0.457 ± 0.0996 Hz; two-tailed *t*-test, *p* < 0.01). However, mPSC amplitude was increased by the treatment of GluA2-G-Gpep (saline: 13.749 ± 0.858 pA; GluA2-G-Gpep: 18.622 ± 0.79 pA; two-tailed *t*-test, *p* < 0.01) (Fig. 8a). In addition, the rise time in neurons treated with GluA2-G-Gpep was significantly enhanced (saline: 1.66 ± 0.075 ms, n = 9; GluA2-G-Gpep: 2.124 ± 0.157 ms, n = 14; two-tailed *t*-test, *p* < 0.01), but there was no difference in decay time between the two groups (Fig. 8a). As shown in Fig. 8b and supplementary Fig. 6, both the field excitatory postsynaptic potentials (fEPSPs) in CA3-CA1 synapses induced by stimuli at varying intensities and the paired-pulse ratio (P2/P1 of fEPSP) at different interstimulus intervals were similar between both mice. Our analysis using one-way ANOVA resulted in F = 0.928 and *p* = 0.356, indicating that there was no significant change in basic synaptic activity at CA3-CA1 synapses between the two groups. Example traces for the paired-pulse ratio are given on the right. However, LTP induced by high frequency stimulation (HFS) in CA3-CA1 synapses of peptide-treated mice was significantly enhanced versus saline (saline: 1.52 ± 0.1, n = 6; GluA2-G-Gpep: 1.79 ± 0.1, n = 8, two-tailed *t*-test, *p* < 0.05) (Fig. 8c). Our data suggest that the interaction between GluA2 and GAPDH during development could be involved in regulating synaptic plasticity in CA3-CA1 synapses.

## Discussion

In this study, we investigated the roles of GluA2-GAPDH interaction in neurodevelopment both *in vitro* and *in vivo*, using an interfering peptide (GluA2-G-Gpep) that is able to specifically disrupt this interaction. We found pronounced defects in axon and dendrite development, as well as fewer neuron numbers, altered cortical lamination and synaptic activity. Our results may represent a novel mechanistic role for the GluA2-GAPDH interaction in AMPAR-mediated early development of axons, dendrites and cortical architecture.

Individual neuron growth requires proper developmental processes in growth cone dynamics, axon integrity, dendritic branching, spine development and synapse connections. From our imaging results, disrupting the GluA2-GAPDH interaction produced abnormalities in all of these stages. Consistent with these findings, Schenk *et al*. has reported that GluA2 subunits are expressed and organized in axonal growth cones of neurons^24^. Overexpressing GluA2 has also been shown to increase dendritic length, arborization and spine density of pyramidal neurons^28^,^29^,^30^, while downregulation results in a reduction of dendritic arborization in early spinal motoneurons^34^. A recent study also provided evidence for the localization of GAPDH with GluA2 and L1 at lens fibre cell membranes, further illustrating the potential involvement of this complex in axon formation^35^. Interestingly, our *in vivo* data indicated that the GluA2-GAPDH interaction may play a more specific role in the development of cortico-cortical than cortico-striatal connections. A study on Alzheimer’s disease has reported that glutamatergic transmission is severely altered by the early degeneration of cortico-cortical connections^36^. This discrepancy could also be explained by the heterogeneous nature of cortico-striatal neurons, where the different subtypes have projections to distinct brain regions and hence their development may be governed by other factors and/or receptors^37^.

The underlying mechanisms in which how the GluA2-GAPDH complex regulates neurodevelopment are likely to be complicated and involved various different pathways. The NTD of GluA2 interacts with N-cadherin and Plexin A4 in regulating spine density and dendritic arbors^16^,^17^. In particular, Yamashita *et al*. has extensively demonstrated GluA2-Plexin A4 interacting signals were detected in cell bodies and dendrites in HEK293 cells, rat brain lysates and cultured neurons^16^. Our co-immunoprecipitation results provide important clues that disrupting GluA2-GAPDH interaction specifically results in GluA2 having a lower binding affinity with Plexin A4, which could be due to altered conformation or semaphorin signaling^16^. On the contrary, dendritic spine development with GluA2-GAPDH complex appears to be unrelated to the interaction between GluA2 and N-cadherin. Interestingly, the increase in GluA2 binding to stargazin may be a result of more GluA2 available from GluA2-GAPDH disruption, and this would have significant effects on AMPAR trafficking, receptor kinetics and synaptic transmission^10^,^38^.

Recent experiments have demonstrated that the transcription factor p53, other than regulating neuronal cell death, is also critical for neurodevelopmental processes, such as neurite outgrowth, axon guidance and regeneration^22^,^23^. The primary determinant of p53-mediated effects depends on its post-translational modification, where p53 acetylation of lysine residues modulates neurodevelopment^23^. We previously reported that glutamate-induced internalization of the GluA2-GAPDH complex allows GAPDH to interact with p53 in promoting neuronal cell death via increased S46 phosphorylation^21^, indicating a relationship between this interaction and p53-mediated effects. The reduced expression of acetylated p53 K320 from uncoupling the GluA2-GAPDH interaction during development further implies that this complex could play an important role in the post-transcriptional modification of p53. More importantly, prior studies have shown a positive correlation between acetylated K320 of p53 and stimulation of neurite outgrowth and branching^22^. Adult mice with peptide treatment displayed similar levels of acetylated K320, corresponding to minimal neurite outgrowth at this stage. In conclusion, the GluA2-GAPDH complex may regulate neurodevelopment at least in part through GluA2 interaction with Plexin A4 and acetylation of K320 in the downstream target p53. More research is necessary to decipher the exact relationship between the GluA2-GAPDH complex with Plexin A4, stargazin and p53, and determine other pathways that are involved.

We extended our analysis to screen for other histological defects *in vivo*, including neuron number, neuronal proliferation and laminar position with GluA2-GAPDH disruption during early neurodevelopment. Surprisingly, we observed fewer neuron numbers, reduced neurogenesis and altered neuron positioning in the cortex. Whitney *et al*. has reported that the unedited Q form of GluA2 is essential in promoting AMPA-mediated differentiation from progenitors to neurons^27^. Thus, blocking GluA2-GAPDH interaction may prevent GluA2 from stimulating neuron differentiation, resulting in fewer neuron numbers. Interestingly, the increase in neuronal proliferation at P1 may represent a compensatory mechanism for rescuing neuron loss at earlier stages. In addition, there is evidence that GluA2 subunits show a distinct laminar distribution pattern in the macaque visual cortex, with various cortical layers differentially influenced by glutamate^39^. But it remains unclear how AMPAR GluA2 subunits are related to neurogenesis and neuronal positioning. Our findings present new evidence that GluA2-GAPDH interaction may potentially be involved in regulating these processes, and provide insights towards future research in understanding the mechanisms involved.

In terms of neuronal function, we found that the reduced spine density may be associated with alterations in mPSC frequency, amplitude and rise time. Consistent with our results, a recent study using the A30P α-SYN transgenic mice displayed impairments in dendritic branching and spine density, corresponding with decreased mPSC frequency and increased mPSC amplitude^40^. Other studies have also demonstrated a positive correlation between spine loss and decreased mPSC frequency, suggesting that synapse number is a strong determinant of neuronal transmission^41^. It is possible that GluA2-GAPDH disruption may decrease the probability of glutamate release in explaining the reduction in mPSC frequency, but future experiments are required to address this issue. In contrast, the increase in mPSC amplitude and rise time may indicate a plausible compensatory effect to spine density reduction, as similar paradoxical effects have been reported with enhanced LTP observed^42^. Moreover, this may be the result of more postsynaptic AMPAR expression since our group has previously shown that the GluA2-G-Gpep can prevent agonist-induced GluA2 internalization^20^. Further research is essential to dissect the complicated relationships with GluA2-GAPDH complex, dendritic spine changes and synaptic transmission in the brain.

Traditionally, the function of ligand-gated ion channels was thought to be modulated only through receptor phosphorylation. However, recent studies have illustrated that protein-protein interactions can also regulate receptor function, trafficking and downstream signaling cascades^43^. More importantly, these interactions have been recognized as putative therapeutic targets for the development of new treatments, such as in stroke and cancer, with several clinical trials ongoing^44^,^45^. Abnormally enhanced GluA2-GAPDH interactions are associated with neuronal cell death, and we have shown that GluA2-G-Gpep administration successfully rescued ischemic stroke and multiple sclerosis mouse models^18^,^19^. However, basal levels of GluA2-GAPDH complex exist in healthy subjects of post-mortem brain tissues, suggesting that this interaction may have important physiological roles^19^. It is unlikely that the observed results presented in this study are caused by a direct effect of our peptide on other GluA subunits, as we have previously reported that GAPDH interacts exclusively with GluA2 and that GluA2-G-Gpep is specific for this interaction^20^. However, other mechanistic pathways such as calcium signaling could indirectly be responsible for how GluA2-GAPDH complex governs neurodevelopment. Until now, evidence is still lacking for the association between diminished GluA2-GAPDH interactions and any mental disorders. Nevertheless, we have presented novel information about the relationship between a specific receptor-protein complex and neurodevelopment, which may ultimately provide mechanistic insights about neurodevelopmental diseases including schizophrenia, that are associated with dysfunctional glutamate signaling; and development of new treatment options with effective functional outcomes.

## Methods

### Mice

Mice on a CD-1 background were bred at the Centre for Addiction and Mental Health (CAMH) (Toronto, Canada). Littermates from the same breeding batch were used for each experiment. All mouse protocols were approved by the CAMH Animal Care Committee and that all methods were carried out in accordance with the approved guidelines.

### GluA2-G-Gpep peptide synthesis

GluA2-G-Gpep (YK-41) peptide was synthesized by Biomatik Corporation (Cambridge, Canada). The cell membrane transduction domain of HIV-1 TAT protein sequence (YGRKKRRQRRR)^46^ was fused to the N-terminus of the peptide, facilitating its intracellular delivery through the placenta and entering embryonic brains^47^. The final protein sequence of GluA2-G-Gpep used in this study was YGRKKRRQRRR-YYQWDKFAYLYDSDRGLSTLQQVLDSAAEK. The peptide was further purified by high-performance liquid chromatography to 98% purity, dissolved in 0.9% saline and aliquots were stored at −80 °C. The TAT-control peptide is comprised of only the TAT protein sequence.

### Primary neuron culture preparation

Cortical tissues from embryonic day 14 (E14) mouse brains were dissected out, incubated with 0.25% trypsin for 15 min at 37 °C, and dissociated by mechanical trituration. Neurons were then plated at a desired density onto glass coverslips previously coated with 0.1 mg/ml poly-d-lysine, and grown in Neurobasal medium with 4 mM L-glutamine, 1× B27, 100 U/ml penicillin and 100 μg/ml streptomycin in an incubator (37 °C, 5% CO_2_) until neurons were suitable for the specified experiments. 10 μM of GluA2-G-Gpep or TAT-control peptide was added into culture plates 12 hours in advance at 3 DIV for growth cone analysis, 8 DIV for axon and dendrite growth, and 12 DIV for spine development.

### GluA2-G-Gpep *in vivo* treatment regimen

5 nmol/g of GluA2-G-Gpep or saline was carefully injected (intraperitoneal) into pregnant female mice daily from E8–19, where neurodevelopment is most prominent. Our group has previously described that chronic daily treatment of this chosen peptide concentration effectively reduces the GluA2-GAPDH interaction^19^.

### Immunofluorescence

#### Immunohistochemistry

Pregnant female mice were sacrificed at either E16 for the analysis of neurogenesis or P1 for all other immunomarkers. Brains were harvested, fixed in 4% paraformaldehyde (PFA) overnight, cryoprotected in 30% sucrose and frozen at −80 °C before further processing. 10 μm-thickness frozen coronal sections were cut using a microtome cryostat system. Free floating sections were initially blocked in 5% fetal bovine serum, 1% Triton X-100, 0.5% Tween 20 and 1% skim milk in 0.1 M PBS for 2 hours at room temperature to reduce non-specific binding. This was followed by incubation with primary antibodies overnight at 4 °C and secondary antibodies for 2 hours in blocking solution at room temperature. The following primary antibodies were used: anti-HIV1 tat (1:200; Abcam, Cambridge, MA, USA), anti-L1 (1:200; Millipore, Bellerica, MA, USA) and anti-2H3 (1:500; Developmental Studies Hybridoma Bank, University of Iowa, IA, USA), anti-NeuN (1:200; Millipore), anti-Ki67 (1:100; Abcam), anti-Cux1 (1:200; Santa Cruz Biotechnology, Dallas, TX, USA), anti-Ctip2 (1:200; Abcam), anti-Plexin A4 (1:200; Abcam) and anti- acetyl-p53 (Lys320) (Millipore). Fluorescent secondary antibodies conjugated to Alexa 488 or 594 (1:200; Life technologies) were used for detection of primary antibodies. Staining of F-actin and nuclei was achieved with Alexa 488 phalloidin and DAPI, respectively.

#### Immunocytochemistry

Cultured neurons were fixed in 4% PFA/4% sucrose, permeabilized with 0.1 M PBS containing 0.1% Triton X-100 for 10 min, and blocked for 1 hour with 1% bovine serum albumin in PBS at room temperature. Similarly, they were incubated with primary antibodies overnight at 4 °C and secondary antibodies for 1 hour at room temperature. Primary antibodies include anti-Tau1 (1:200; Millipore), anti-TuJ1 (1:500; Millipore and Abcam) and anti-MAP2 (1:500; Millipore).

### Time-lapse video imaging

Primary neuronal cultures were plated on 35 mm glass bottom dishes from MatTek Corporation (Ashland, MA, USA). Differential interference contrast (DIC) images were captured using the Vivaview FL incubator microscope (Olympus, Toronto, Canada). The recording parameters were set at every 2 minute for 24 hours with growth cone analysis experiments, while images were taken at 10-minute intervals for 60 hours for the analysis of dendritic branching.

### Immunostaining analysis

#### Immunocytochemistry

All fluorescent images were captured using a confocal microscope (Olympus FluoView FV1200) at 60× magnification for neurite development, and 100× for growth cone and spine density analysis. The number of filopodia and growth cone area was analyzed from both axonal and dendritic ends. For Tau1 staining of axons, additional heat maps of fluorescent intensity were used to define axonal tract integrity. Sholl analysis was utilized to provide a quantitative measure of the radial distribution of neuronal dendritic arborization^48^. Using Image J (http://imagej.nih.gov/ij/), 10 concentric and equidistant circles (3.5 μm separation of each radius) were constructed and centered at the perikaryon of each neuron. The number of dendritic intersections crossing each increasing radius was calculated. Moreover, the log of the number of intersections per circle area versus circle radius was plotted, in which the slope of the regression line (Sholl regression coefficient) represents a measure of the decay rate of dendritic branches with distance from the soma^48^. The Schoenen ramification index (maximum number of intersections/number of primary dendrites), a measure of the ramification richness for each neuron^49^, and the number of dendritic bifurcations all provide important information on the degree of dendritic complexity. Finally, dendrites with distinct spine protrusions were counted and spine density was expressed as the number of spines per dendritic length (μm).

#### Immunohistochemistry

Fluorescent images were captured at 10× magnification using the Zeiss LSM510 Meta confocal microscope, converted to grey-scale and normalized to background staining. Sections chosen for analyses were anatomically-matched between comparing groups, and included samples from rostral to caudal regions. A two-dimensional random sampling window approach on regions of interest (ROI) was employed to provide accurate estimates of cell densities and fluorescent occupancies^50^. Fluorescent cells within each ROI were counted using the ITCN plugin for ImageJ. As for fluorescent occupancies, images were converted to a pre-calibrated black and white threshold scale using ImageJ, in which fluorescent intensities that reach a standard threshold become black while the rest remain white. Therefore, quantification of axonal tract loss was measured as the percentage of area occupied by fluorescent-labeling in each ROI. Mean grey values were also used to define axon integrity (Image J). The width of cortical tracts was measured in consistent ROIs, matching corresponding positions. All image-capturing and threshold parameters were kept the same for each measurement between comparing groups. ROIs of fixed area were positioned over various cortical regions for each analysis. Specifically for Cux1 and Ctip2 antibodies, each ROI was further subdivided into eight equal regions from the pia to the inner border of the cortex, to assess neuron distribution across the layers of the cortex (Supplementary Fig. 2b). The distribution was expressed as a percentage of the numbers of labeled-cells in each bin divided by the total numbers within each ROI.

### Co-immunoprecipitation, protein affinity purification and Western blot

Co-immunoprecipitation, protein affinity purification and Western blot procedures were performed according to previous methods^51^,^52^. For co-immunoprecipitation, 500–700 μg solubilized protein was extracted from E16 brains and incubated in the presence of anti-GluA2 (Novus Biologicals, Oakville, Canada) or control IgG (1–2 μg) for 4 hours at 4 °C, followed by the addition of protein A/G plus agarose (Santa Cruz Biotechnology) for 12 hours. For affinity purification experiments, 50–100 μg of protein was incubated with glutathione-sepharose beads (GE Healthcare Life Sciences, Mississauga, Canada) bound to the indicated GST-fusion proteins (50–100 μg) at room temperature for 1 hour. GST-GluA2-G-Gpep was prepared as previously described^20^, and GST-FARP2 (1–350) was purchased (Abnova Taiwan Corporation, Taiwan). Both pellets and beads were washed, boiled in sodium dodecyl sulfate (SDS) sample buffer for 5 min and subjected to SDS-polyacrylamide gel electrophoresis. Proteins were subsequently transferred onto nitrocellulose membranes and Western blotted with anti-GluA2 (Millipore), anti-GAPDH (Millipore), anti-N-cadherin (BD bioscience, Mississauga, Canada), anti-Plexin A4 (Abcam), anti-stargazin (Millipore), anti-Narp (NPTX2) (Abcam) antibodies. Similarly, Western blot analysis was performed on P1 and adult brain tissues of both groups. The antibodies used were anti-acetyl-p53 (Lys320) (Millipore) and anti-acetyl-p53 (Lys382) (Cell Signaling Technology, Beverly, MA, USA). The intensity of all resulting bands was quantified by densitometry using Image J. Co-immunoprecipitation bands were normalized to GluA2 immunoprecipitation bands and expressed as the percent of saline controls.

### Golgi-Cox Staining

Golgi-Cox staining was performed as described previously^53^. Briefly, newborn mice of both groups were raised until 6 weeks old. They were anesthetized with xylazene/ketamine (10 ml/kg) and intracardially perfused with 0.9% saline. Brains were removed and immersed in Golgi-Cox solution for 2 weeks before transferring to 30% sucrose solution for 5 more days. 200 μm-thick sections were sliced using a microtome (Leica VT1000S, Concord, Canada) for further staining and fixation. Golgi-stained images captured at 100× magnification were used for analysis of spine density (Nikon Eclipse E600, Melville, New York, USA). All spine types were counted only on the apical dendrites initial segment of pyramidal neurons in layers III and V of the frontal cortex. Spine density was expressed as the number of spines per dendritic length (μm).

### Electrophysiology

Age-matched adult mouse brains (16 weeks old for mPSC, 6 weeks old for fPSP) were dissected out and placed in ice-cold oxygenated artificial cerebrospinal fluid (aCSF) (95% O_2_, 5% CO_2_) containing 124 mM NaCl, 3 mM KCl, 1.25 mM NaH_2_PO_4_, 1.3 mM MgCl_2_, 2.6 mM CaCl_2_, 26 mM NaHCO_3_ and 10 mM glucose. Hippocampal coronal slices of 350 μm thickness were cut using a microtome (Leica VT1000S). For miniature current recording, the animals were intracardially perfused with cold oxygenated cutting solution (220 nM sucrose, 2.5 nM KCl, 1.25 mM NaH_2_PO_4_, 25 mM NaHCO_3_, 0.5 mM CaCl_2_, 7 mM MgCl_2_ and 20 mM D-glucose). After 1 hour recovery period in oxygenated aCSF, slices were transferred to a recording chamber continuously perfused with oxygenated aCSF (3 ml/min) and kept at approximately 30–31 °C. For mPSC recordings, tetrodotoxin (TTX, 0.1 μM) was added into the aCSF and membrane potential was kept at −60 mV using the whole cell configuration. The recording pipette was pulled from borosilicate glass and showed a resistance of 4–6 MΩ after filling with intracellular solution (132.5 mM Cs-gluconate, 17.5 CsCl, 10 mM HEPES, 0.2 mM EGTA, 2 mM Mg-ATP and 0.3 mM GTP at pH 7.25 and 290 mOsm). At least 5 min were recorded for each neuron, independent of mini event number. mPSCs were analyzed with miniAnalysis (Synaptosoft Inc, GA USA, Demo version).

fEPSPs were evoked every 30 seconds by electrical stimulation delivered to the Schaffer-collateral pathway via a concentric bipolar stimulating electrode and recorded with glass microelectrodes. The stimuli intensity was adjusted to evoke fEPSPs corresponding to 30~50% of the maximal response evoked in the absence of a contaminating spike discharge. The paired-pulse ratio was determined by delivering pairs of stimuli at varying interstimulus intervals. The fEPSPs were monitored for 20 minutes to ensure baseline stability, followed by induction of synaptic plasticity with HFS (100 pulses at 100 Hz in 1 s. repeated three times at 30 s intervals). fEPSPs were measured for 1 hour, with signals amplified (Axopatch 200B), recorded (Digidata 1322A) and analyzed using Clampfit 10 (Molecular Devices, Sunnyvale, CA, USA).

### Statistical analysis

Statistical differences between no treatment/saline and GluA2-G-Gpep treatment groups were analyzed using one-way ANOVA (GraphPad 5.0) for continuous variables (neuronal distribution, Sholl analysis), followed by Bonferroni’s correction for multiple testing; while the Student’s two-tailed *t*-test was performed for single parameters. All images were blinded prior to analysis. Data were expressed as mean ± standard error of mean. A significance level of *P* < 0.05 was used for all analyses.

## Additional Information

**How to cite this article**: Lee, F. H. F. *et al*. Disrupting GluA2-GAPDH Interaction Affects Axon and Dendrite Development. *Sci. Rep*. **6**, 30458; doi: 10.1038/srep30458 (2016).

## Supplementary Material

Supplementary Information
