# Supplementary material for: Disrupting GluA2-GAPDH Interaction Affects Axon and Dendrite Development
Source: Sci Rep. 2016 Jul 27;6:30458. doi: 10.1038/srep30458 (PMC4962050; doi:10.1038/srep30458)
Supplement: Supplementary Information [file srep30458-s1.pdf]

Disrupting GluA2-GAPDH Interaction Affects Axon and Dendrite Development

[1]Frankie Hang Fung Lee, [1]Ping Su, [1]Yu Feng Xie, [1]Kyle Ethan Wang, [2]Qi Wan and [1,3]Fang Liu

[1]Campbell Research Institute, Centre for Addiction and Mental Health, Toronto, Ontario, Canada. M5T 1R8
[2]Department of Physiology, School of Medicine, Wuhan University, Wuhan 430071, China.
[3]Department of Psychiatry, University of Toronto, Toronto, Ontario, Canada. M5T 1R8

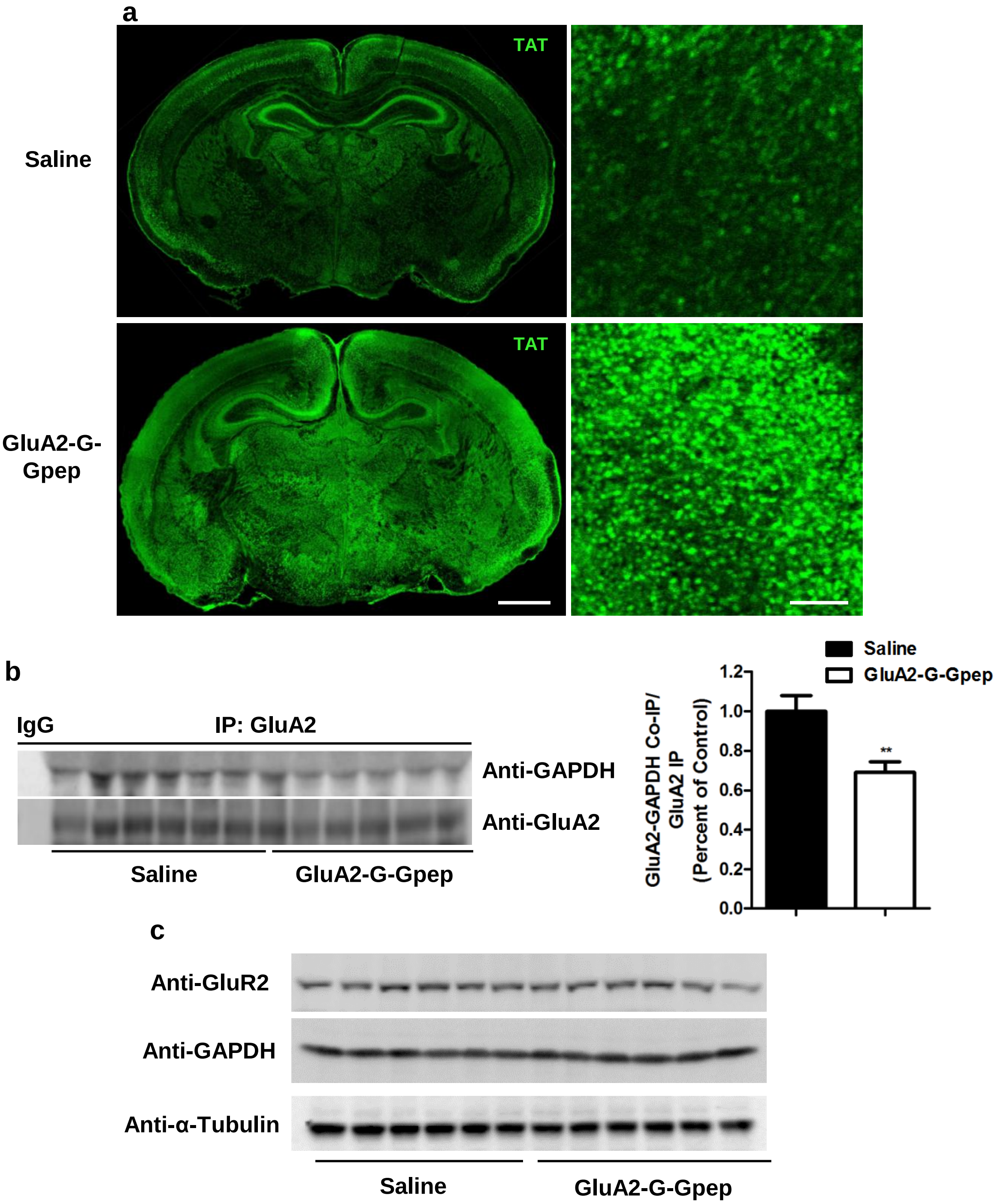

Supplementary Fig. 1. GluA2-G-Gpep injection into pregnant mice was able to pass through the placenta and exert its effect in disrupting GluA2-GAPDH interaction in embryonic mouse brains. (a) Immunohistochemistry using anti-TAT antibody was performed on P1 coronal mouse sections to examine the presence of TAT-GluA2-G-Gpep. Immunofluorescent images from GluA2-G-Gpep-injected brain sections showed stronger fluorescent signals than in control brains, indicating that a significant concentration of GluA2-G-Gpep peptide has entered the neonatal brains (n=9 sections from 3 brains per group). Scale bar: 300µm (left), 100µm (right). **(b)** Coimmunoprecipitation experiments showed that there was a significantly lower level of GluA2-GAPDH complex in E16 embryonic brains of GluA2-G-Gpep-treated pregnant dams (n=6 brains per group; two-tailed *t*-test). **(c)** Total expression levels of GluR2 and GAPDH were similar between the two groups. α-tubulin was used as a loading control. Data are presented as mean ± SEM. ** $p < 0.01$.

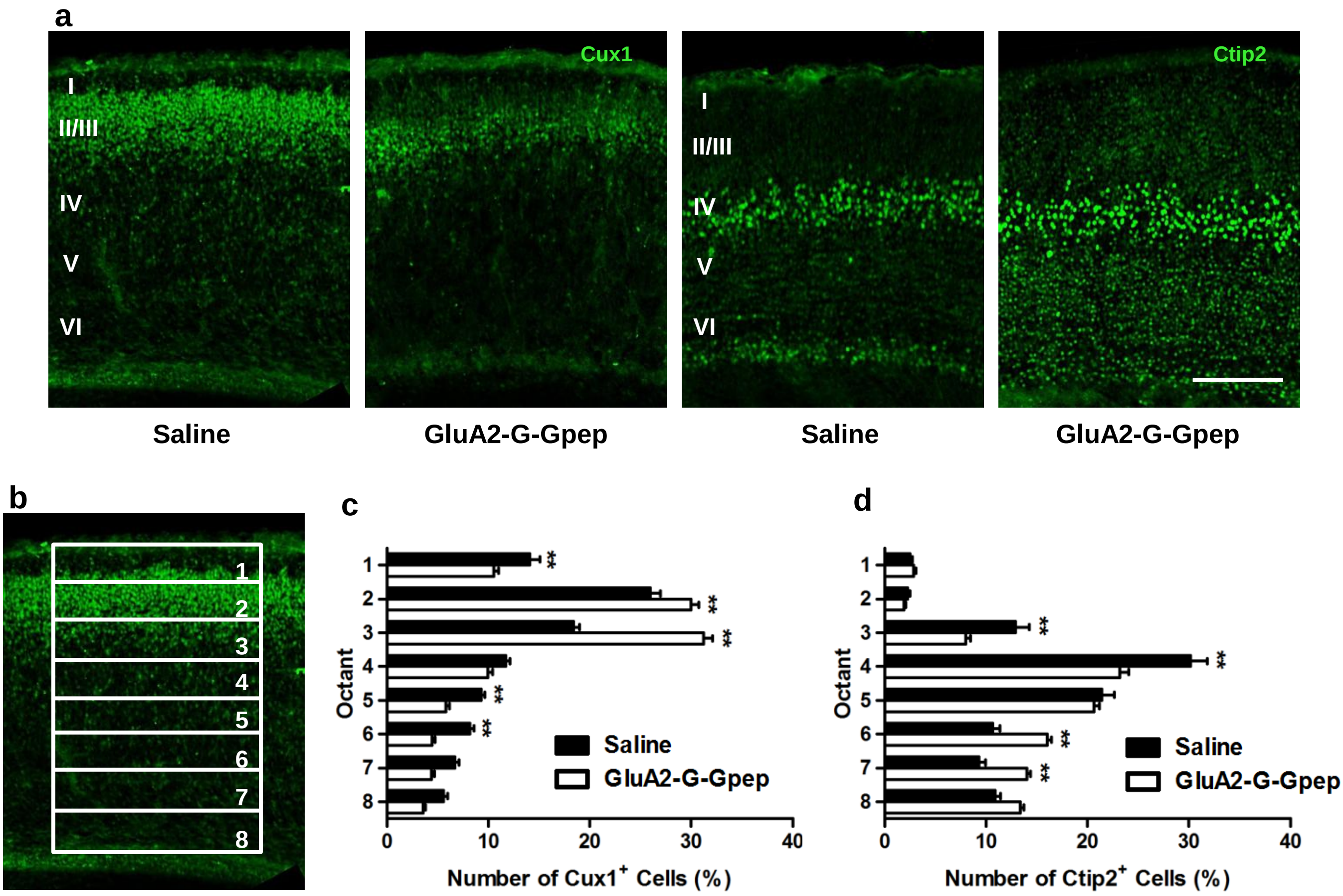

Supplementary Fig. 2. Altered cortical neuron distribution in P1 mouse brains with GluA2-G-Gpep treatment. (a) Representative images of Cux1- and Ctip2-fluorescently labeled cells, specific for layers II/III and layers IV respectively, in saline and GluA2-G-Gpep-treated mice. Scale Bar: 100μm. **(b)** A rectangular ROI spanning the width of the cortex was subdivided into eight equal octants, in which the number of Cux1 and Ctip2 cells was counted and expressed as a percentage of total cells in each ROI. **(c)** GluA2-G-Gpep-treated mice displayed a significantly higher proportion of $Cux1^{+}$ cells at superficial layers of octants 2 and 3, while for saline groups, $Cux1^{+}$ cells were more evenly distributed with higher percentages in middle cortical layers of octants 5 and 6 (saline: n=36; GluA2-G-Gpep: n=48 ROIs from 4 different brains; one-way ANOVA, *post hoc* Bonferroni correction). **(d)** A significantly larger proportion of $Ctip2^{+}$ cells was found within deeper layers of octants 6 and 7 in GluA2-G-Gpep-treated mice, but those in controls were focused near the middle of octant 4 (saline: n=42; GluA2-G-Gpep: n=51 ROIs from 4 different brains; one-way ANOVA, *post hoc* Bonferroni correction). Data are presented as mean ± SEM. ** $P < 0.01$. ROI - region of interest; I-VI - cortical layers.

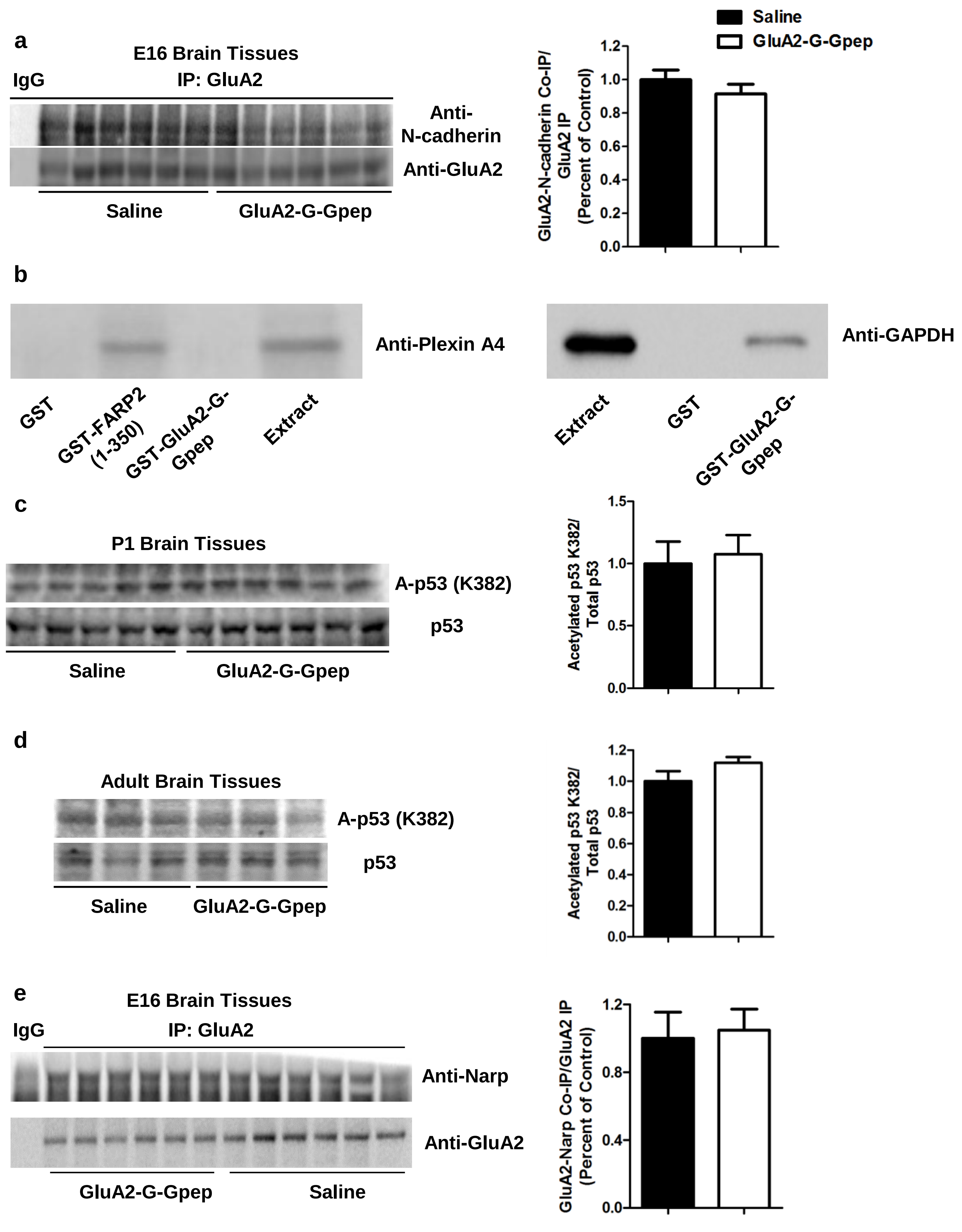

Supplementary Figure 3 Lee *et al.* (2016)

Supplementary Fig. 3. Disrupting GluA2-GAPDH interaction did not alter GluA2-N-cadherin interaction or acetylated Lys382 p53 expression levels in mouse brains at different ages. (a) Co-immunoprecipitation of E16 brains between GluA2 and N-cadherin showed no differences in their interaction between saline and GluA2-G-Gpep treatment groups (n=6 brains per group; two-tailed *t*-test). **(b)** Glutathione S-transferase (GST) pull-down assay using GST-GluA2-G-Gpep indicated a direct interaction between our peptide and GAPDH, but not with Plexin A4. GST-FARP2 (1-350) was used as a positive control that interacts with Plexin A4. Western blots of acetylated lysine 382 (K382) of p53 in **(c)** P1 and **(d)** adult mouse brains. Acetylated K382 p53 expression levels were similar between saline and peptide-treatment groups at both stages (P1 – saline: n=5, GluA2-G-Gpep: n= 6 brains; Adult – n=3 brains per group; two-tailed *t*-test). **(e)** There was no significant difference in GluA2-Narp interaction with GluA2-G-Gpep treatment as well (n=6 brains per group; two-tailed *t*-test). Quantification of GluA2 interaction was calculated by normalizing to GluA2 immunoprecipitation bands and expressing as percent of saline controls. Data are presented as mean ± SEM.

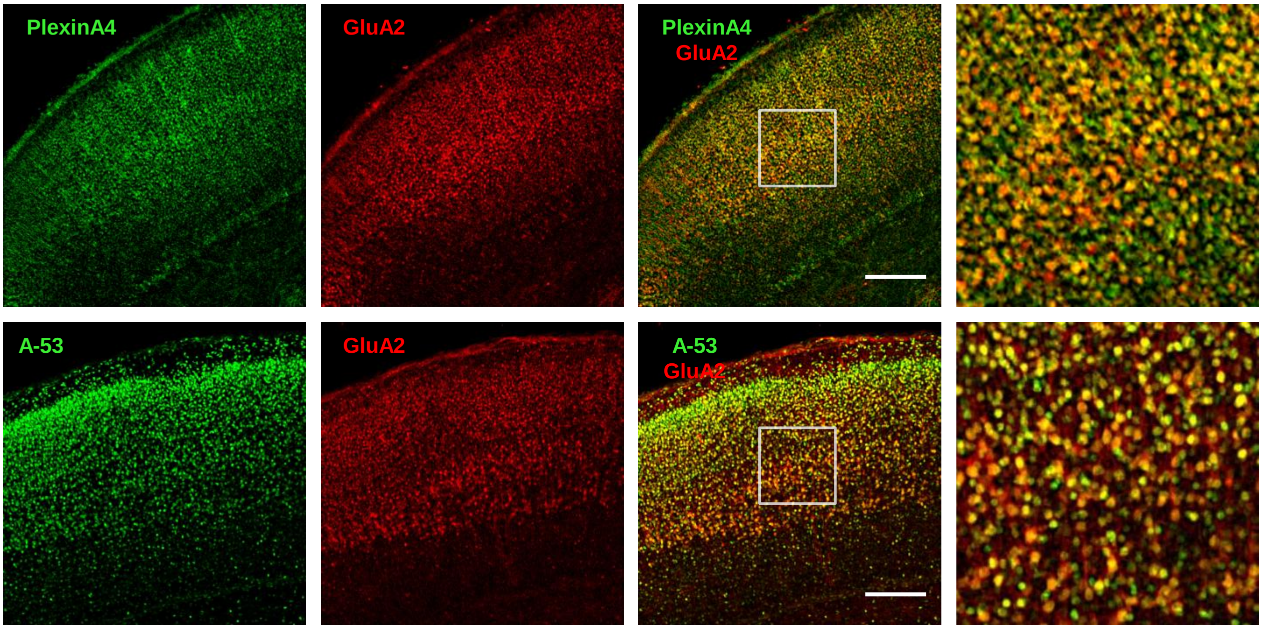

Supplementary Fig. 4. GluA2, Plexin A4 and acetylated p53 (Lys320) are present in the cortex of developmental mouse brains. Immunohistochemistry using antibodies against Plexin A4 and acetylated p53 (Lys320) with GluA2 in P1 mouse brain sections showed that GluA2, Plexin A4 and acetylated p53 (Lys320) are present in close proximity within the cortex. Scale Bar: 100µm.

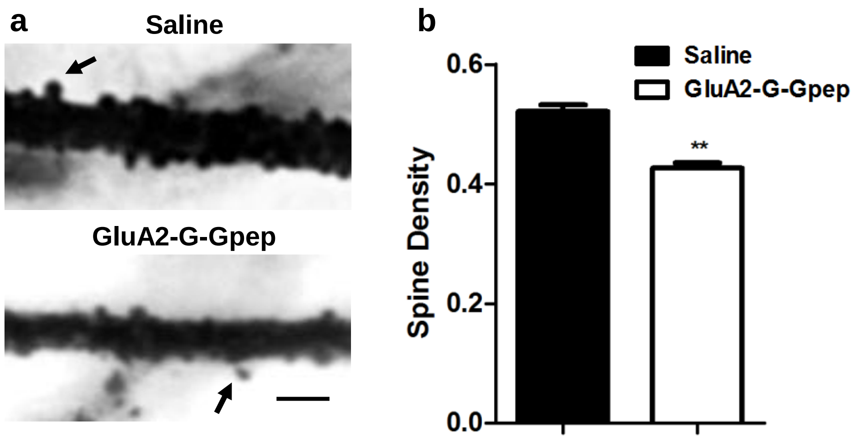

Supplementary Fig. 5. GluA2-G-Gpep treatment during early development produces spine deficits in cortical pyramidal neurons of older mice. (a) Golgi-stained images of dendritic spines (arrows) of cortical pyramidal neurons in control and GluA2-G-Gpep-treated mice are shown. **(b)** Early developmental treatment with GluA2-G-Gpep resulted in a significant reduction of dendritic spine density compared to saline group (saline: n=78 neurons from 4 different brains; GluA2-G-Gpep: n=88 neurons from 4 different brains; two-tailed *t*-test). Scale Bar: 5µm. Data are presented as mean ± SEM. ** $p < 0.01$.

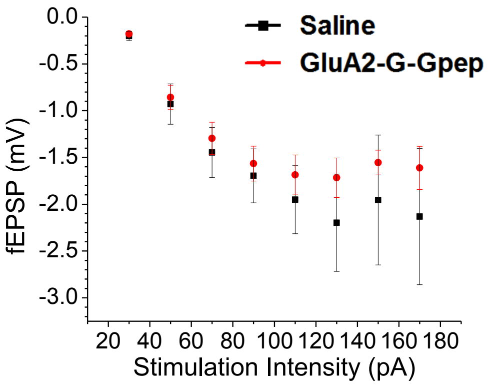

Supplementary Fig. 6. No change in field excitatory postsynaptic potentials (fEPSPs) in CA3-CA1 synapses induced by varying stimulation intensities between saline and GluA2-G-Gpep-treated mice.
